# Opposing profiles of Netrin-1 and adiponectin in metabolic inflammation and insulin resistance

**DOI:** 10.3389/fphar.2025.1632956

**Published:** 2025-08-28

**Authors:** María Guadalupe Ramos-Zavala, Jesús Jonathan García-Galindo, Alberto Beltrán-Ramírez, Luis Ricardo Balleza-Alejandri, Emiliano Peña-Durán, Alfredo Huerta-Huerta, Edy David Rubio-Arellano, Luis Daniel López-Murillo, Sara Pascoe-Gonzalez, Tannia Isabel Campos-Bayardo, Daniel Osmar Suárez-Rico

**Affiliations:** ^1^ Departamento de Fisiología, Centro Universitario de Ciencias de la Salud (CUCS), Universidad de Guadalajara, Guadalajara, Mexico; ^2^ Institute of Experimental and Clinical Therapeutics (INTEC), Health Science University Center, Universidad de Guadalajara, Guadalajara, Mexico; ^3^ Instituto de Investigación en Ciencias Biomédicas, Centro Universitario de Ciencias de la Salud, Universidad de Guadalajara, Guadalajara, Jalisco, Mexico; ^4^ Hospital Médica de la Ciudad, Guadalajara, Mexico; ^5^ Licenciatura en Médico Cirujano y Partero, Centro Universitario de Ciencias de la Salud (CUCS), Universidad de Guadalajara, Guadalajara, Mexico; ^6^ División de Medicina Molecular, Laboratorio de Microbiología Molecular II, Centro de Investigación Biomédica de Occidente (CIBO), Guadalajara, Mexico

**Keywords:** netrin-1, adiponectin, insulin resistance, obesity, inflammation, macrophages, Hs-CRP, IL-6

## Abstract

**Introduction:**

Obesity-driven low-grade inflammation contributes to insulin resistance (IR) and metabolic dysfunction. Beyond its axon-guidance role, Netrin-1 promotes macrophage retention in inflamed adipose tissue, whereas adiponectin exerts anti-inflammatory, insulin-sensitizing effects. We aimed to compare serum Netrin-1, interleukin-6 (IL-6), high-sensitivity C-reactive protein (hs-CRP), and adiponectin across metabolic profiles and assess their associations with systemic inflammation.

**Methods:**

We conducted an analytical cross-sectional study in adults (n = 60): metabolically healthy controls (C, n = 20), preclinical obesity (PO; HOMA‐IR < 2.5, n = 20), and clinical obesity with insulin resistance (CO; HOMA-IR>2.5, n = 20). Anthropometrics, fasting glucose/insulin, lipid profile, and serum biomarkers were obtained; group differences and correlations were analyzed (non-parametric tests where appropriate).

**Results:**

Between-group testing showed higher Netrin‐1 in CO versus controls (Kruskal–Wallis p = 0.013; C < CO), with no significant difference versus PO. IL-6 was elevated in both PO and CO versus controls (Kruskal–Wallis p < 0.001). hs-CRP peaked in PO versus both C and CO (Kruskal–Wallis p < 0.001). Adiponectin was lower in CO and inversely correlated with hs-CRP (r = –0.22) and IL‐6 (r = –0.53, p < 0.001).

**Discussion:**

Circulating Netrin-1 and adiponectin display opposing profiles aligned with pro- versus anti-inflammatory signaling in metabolic dysfunction. These findings support the Netrin–1/adiponectin axis as an immunometabolic marker set in early IR states. The cross-sectional design limits causal inference; longitudinal studies integrating tissue-level measurements are warranted.

## Introduction

Obesity is recognized as a chronic metabolic disease characterized by a state of low-grade systemic inflammation, which plays a fundamental role in the progression of insulin resistance and associated metabolic dysfunctions. This inflammation is primarily driven by the infiltration of immune cells into adipose tissue and the sustained release of proinflammatory cytokines such as interleukin-6 (IL-6) and high-sensitivity C-reactive protein (hs-CRP) (Nedeva et al., 2020; Arivazhagan et al., 2020).

At the molecular level, expanded adipose tissue in obesity experiences relative hypoxia, activating signaling pathways such as HIF-1α and NF-κB, which in turn promote the production of cytokines and chemokines (Poblete et al., 2020; Geiger et al., 2023). This environment induces the recruitment of circulating monocytes and the polarization of macrophages toward a proinflammatory phenotype characterized by the expression of TNF-α, IL-6, and oxidative stress molecules (Ziegon and Schlegel, 2022). Obesity is characterized by chronic low-grade inflammation within adipose tissue, driven by the dysregulation of adipocyte-derived mediators known as adipokines. These molecules orchestrate metabolic and inflammatory responses, contributing to systemic insulin resistance (Lu et al., 2020; Reynoso-Roa et al., 2025).

Netrin-1, recently recognized as a potent immunomodulator within adipose tissue, specifically, promotes macrophage retention through its receptor UNC5B, mediated by the cytoskeletal reorganization, perpetuating local adipose tissue inflammation through nuclear factor-kappa B (NF-κB) signaling pathway and impairing systemic metabolic homeostasis (Ramkhelawon et al., 2014; Sharma et al., 2019; Yang et al., 2023; Jiao et al., 2020). Converging preclinical evidence reinforces this mechanism: in diet-induced obesity, adipose tissue macrophages upregulate Netrin-1 and, through Unc5b, become pathologically retained within adipose depots, perpetuating chronic inflammation and insulin resistance; conversely, myeloid-specific deletion of Netrin-1 facilitates macrophage egress, attenuates adipose inflammation, and improves insulin sensitivity (Ramkhelawon et al., 2014; Sharma et al., 2019; Hsu et al., 2021). *In vitro*, palmitate induces Netrin-1 in macrophages, blunting their migratory capacity and amplifying inflammatory signaling, in line with this retention paradigm (Ramkhelawon et al., 2014). In animal models, changes in insulin sensitivity can affect Netrin-1 and its receptor expression, potentially impacting neural and metabolic development (Batra et al., 2023).

Clinically, circulating netrin-1 appears to behave as a context-dependent marker of metabolic inflammation. Several studies report higher serum netrin-1 in individuals with obesity, prediabetes, or newly diagnosed type 2 diabetes, with positive correlations to insulin-resistance indices (e.g., HOMA-IR) and glycemic markers (Shalaby et al., 2021; Yim et al., 2018; Akbaba E. et al., 2021). In contrast, reduced levels have been observed in more advanced diabetes or β-cell dysfunction, suggesting stage-dependent regulation, differences in inflammatory tone, or exhaustion/adaptation of the netrin-1 axis (Usha et al., 2023; Liu et al., 2016). Collectively, these data position netrin-1 as a dynamic immunometabolic cue whose circulating levels may mirror adipose immune activity and the evolving metabolic state.

In contrast, adiponectin, another adipokine secreted predominantly by healthy adipocytes, mediates potent anti-inflammatory and insulin-sensitizing effects. Its actions involve the activation of AMP-activated protein kinase (AMPK) and inhibition of NF-κB, thereby attenuating local inflammation and enhancing insulin sensitivity (Ouchi et al., 2011). While these two adipokines exhibit opposite immunometabolism functions, it remains unclear how their reciprocal regulation may influence the shift from metabolically healthy obesity towards overt insulin resistance (Tilg et al., 2025). Clarifying these mechanisms could help delineate novel pathways and potential therapeutic targets to prevent obesity-induced metabolic disorders.

In murine models of obesity, the overexpression of netrin-1 in adipose tissue macrophages is associated with impaired emigration, enhanced tissue inflammation, which leads to an increased insulin resistance (Arivazhagan et al., 2020; Sharma et al., 2019). It has been demonstrated that adipokines such as adiponectin play a crucial role in regulating inflammation and metabolic homeostasis. In obesity, reduced adiponectin levels contribute to intracellular activation of inflammatory pathways, such as NF-κB signaling (Akbaba G. et al., 2021).

Recent frameworks, such as those proposed by the Lancet Diabetes & Endocrinology Commission (2025), advocate for a more nuanced categorization that distinguishes between “preclinical obesity” — characterized by increased adiposity without biochemical insulin resistance—and “clinical obesity,” in which adiposity coexists with metabolic dysfunction (Rubino et al., 2025). This distinction becomes particularly relevant when interpreting early glycemic changes. Individuals with clinical obesity often exhibit elevated insulin and HOMA-IR indices, yet maintain fasting glucose within the normal range or even slightly below that of preclinical individuals, due to compensatory hyperinsulinemia. Acknowledging this physiological mechanism is essential to avoid misinterpretation of apparently normal glucose values in the context of insulin resistance.

Thus, obesity generates a complex molecular disruption where netrin-1, IL-6, hs-CRP, and adiponectin act as key modulators of chronic inflammation and early metabolic dysfunction. In this context, the present study aimed to evaluate the serum concentrations of netrin-1, IL-6, hs-CRP, and adiponectin in individuals with different metabolic profiles—non-obese controls, preclinical obese individuals, and clinical obese individuals with insulin resistance—to explore the association between these biomarkers, systemic inflammation, and early metabolic alterations.

## Materials and methods

This analytical cross-sectional study was conducted between 2023 and 2024 at the Instituto de Terapéutica Experimental y Clínica (INTEC), located in the Centro Universitario de Ciencias de la Salud at the Universidad de Guadalajara.

A total of 60 participants were enrolled in the study. The control group (C, n = 20) comprised individuals aged 18–35 years with body-fat percentages <25% in men and <35% in women and a body mass index (BMI) <25 kg/m^2^. The remaining 40 participants met all of the following criteria: (a) age 18–60 years; (b) body-fat percentage >25% in men and >35% in women; and (c) unrestricted physical activity with a monitored caloric intake of 1,800–2,400 kcal/day. These subjects were classified according to their Homeostatic Model Assessment of Insulin Resistance (HOMA-IR) score into preclinical obesity (PO, HOMA-IR <2.5; n = 20) and clinical obesity with insulin resistance (CO, HOMA-IR > 2.5; n = 20). Exclusion criteria included hepatic failure, chronic kidney disease, diagnosis or treatment for coronary, endocrine (including prediabetes, defined as fasting glucose ≥100 mg/dL or HbA1c ≥ 5.7%), rheumatic, or neoplastic disorders, acute infectious diseases, use of anti-inflammatory drugs, dietary supplements, or herbal medicines, as well as pregnancy, breastfeeding, or a confirmed or suspected diagnosis of COVID-19. Participants were also excluded if they reported the use of anti-inflammatory medications (including corticosteroids, non-steroidal anti-inflammatory drugs [NSAIDs], or COX-2 inhibitors), dietary supplements, or herbal products within the previous 30 days. Additionally, to minimize the influence of lifestyle-related confounders on adipokine profiles, participants were required to be non-smokers, report no regular alcohol consumption, and have no dietary restrictions or recent changes in caloric intake. All individuals reported comparable levels of physical activity, and the inclusion criterion of “no limitations in physical activity” ensured functional homogeneity across groups.

Venous blood samples were collected in the early morning after at least 8 h of fasting using red-capped vacuum tubes. Samples were centrifuged at 3,000 revolutions per minute (rpm) for 10 min, and the serum was separated and stored at −80 °C until further analysis. Anthropometric measurements, including weight, height, body mass index (BMI), abdominal circumference, and body fat percentage, were obtained using standardized protocols. BMI was calculated as weight in kilograms divided by the square of height in meters. Body composition was assessed by bioelectrical impedance analysis using a TANITA™ TBF-215 GS device. Systolic and diastolic blood pressure were measured in triplicate using an automated sphygmomanometer following a 10-min rest period. Fasting glucose, serum insulin, lipid profile (total cholesterol, HDL, LDL), and inflammatory biomarkers were evaluated in all subjects. HOMA-IR was calculated using the formula: fasting insulin (μIU/mL) × fasting glucose (mg/dL)/405 nm.

Serum concentrations of IL-6, insulin, adiponectin, netrin-1, and high-sensitivity C-reactive protein (hs-CRP) were quantified using sandwich-type enzyme-linked immunosorbent assays (ELISAs), following the manufacturers’ instructions for each assay. IL-6 were measured using the LEGEND MAX™ ELISA kits (BioLegend, United States), while insulin was assessed with the Human Insulin ELISA kit (Grupo Mexlab, Cat. No. 6001011), and adiponectin was determined with the Human Adiponectin ELISA Kit (MyBioSource, Cat. No. MBS162875). Netrin-1 was measured using the Human Netrin-1 ELISA Kit (MyBioSource, Cat. No. MBS700176), and hs-CRP levels were determined with an ELISA kit from MP Biomedicals (Solon, OH, United States, Cat. No. 07BC-1119). For the determination of netrin-1, samples were activated with 1 N HCl and neutralized using 1.2 N NaOH/0.5 M HEPES buffer, in order to convert the latent form of the molecule to its immunoreactive state. All ELISA measurements were performed duplicate, and absorbance was read at 450 nm using a microplate reader. Concentrations were calculated using four-parameter logistic (4PL) regression curves generated from the standards of each kit. To ensure the integrity of the samples, repeated freeze-thaw cycles were avoided, and all incubations were performed under controlled temperature conditions. Statistical analysis was conducted using R software version 4.1.2 (R Core Team, 2021). Normality of continuous variables was assessed using the Kolmogorov–Smirnov test. Pairwise comparisons were performed with Wilcoxon signed-rank tests, applying Holm correction as the *post hoc* procedure after a Kruskal–Wallis test with Bonferroni adjustment for multiple hypotheses. Correlation analyses were conducted using Spearman’s rank correlation coefficient, and 95% confidence intervals were calculated intervals. Results were expressed as medians and interquartile ranges, or as means ± standard deviation, as appropriate. A p-value ≤0.05 was considered statistically significant.

## Results

Table 1 describes the anthropometric and metabolic characteristics of the study population. Sex distribution did not show statistically significant differences between the groups (p = 0.052); however, a higher proportion of women was observed in the CO group compared to the other groups.

**TABLE 1:** Anthropometric and metabolic characteristics of the study population.

Variable	Control (n = 20)^a^	Preclinical Obesity (n = 20)^a^	Clinical Obesity (n = 20)^a^	p-value^b^
Sex				0.052
Men	10 (50%)	11 (55%)	4 (20%)	
Women	10 (50%)	9 (45%)	16 (80%)	
Age (years)	21 (20–32)	24 (21–57)	28 (20–58)	0.5
Height (cm)	168 (160–172)	170 (164–178)	161 (153–172)	0.034
Weight (kg)	68 (57–77)	94 (87–100)	81 (75–96)	<0.001
BMI (kg/m^2^)	23.8 (21.6–26.7)	32.3 (31.6–33.8)	31.4 (30.2–33.0)	<0.001
Body Fat %	21 (19–24)	32 (28–36)	33 (32–38)	<0.001
Abdominal Circumference (cm)	81 (71–91)	107 (100–111)	105 (99–110)	<0.001
HOMA-IR	1.9 (1.4,-2.4)	2.2 (1.8–2.4)	7.7 (5.8–10.2)	<0.001

^a^ n (%); Values are presented as median and interquartile range (Q1, Q3).

^b^ Kruskal–Wallis rank sum test.

Anthropometric variables demonstrated clinically relevant differences. Body weight was significantly higher in the PO group (mean = 94 kg, range = 87–100) compared to the CO group (mean = 81 kg, range = 75–96), and the C group (mean = 68 kg, range = 57–77) (p < 0.001). Consistently, the body mass index (BMI) was higher in the PO group (mean = 32.3 kg/m^2^, range = 31.6–33.8 kg/m^2^) and the IR group (mean = 31.4 kg/m^2^, range = 30.2–33.0 kg/m^2^) compared to the C group (mean = 23.8 kg/m^2^, range = 21.6–26.7 kg/m^2^) (p < 0.001).

The percentage of body fat showed a statistically significant difference (p < 0.001) in the CO group compared to the rest of the population of the study, suggesting an overall increase in adiposity as part of the metabolic phenotype. Similarly, abdominal circumference was significantly larger in the PO and CO groups compared to the C group (p < 0.001), indicating central fat distribution, a key factor in metabolic complications.

Finally, insulin resistance, assessed through the HOMA-IR index, was significantly higher in the CO group (mean = 7.7, range = 5.8–10.2) compared to the C group (mean = 1.9, range = 1.4–2.4) and the PO group (mean = 2.2, range = 1.8–2.4) (p < 0.001). This difference confirms the presence of an underlying metabolic dysfunction in the CO group, which may contribute to the systemic inflammatory state characteristic of insulin resistance.

Table 2 presents the clinical and metabolic parameters of the study subjects, showing there is no significant difference in systolic blood pressure (SBP) among the groups (p = 0.3), whereas diastolic blood pressure (DBP) showed higher levels in the PO group compared to the CO and C groups (p = 0.033), suggesting a potential impact of obesity on blood pressure regulation.

**TABLE 2:** Clinical and metabolic parameters of the study population.

Variable	Control (n = 20)^a^	Preclinical Obesity (n = 20)^a^	Clinical obesity (n = 20)^a^	p-value^b^
SBP (mmHg)	118 (107–124)	121 (113–126)	120 (115–124)	0.3
DBP (mmHg)	72 (71–80)	79 (76–84)	77 (73–80)	0.033
Glucose (mg/dL)	76 (68–89)	92 (79–112)	87 (75–118)	0.10
Insulin (μIU/mL)	8 (7–14)	9 (7–12)	39 (26–48)	<0.001
Total Cholesterol (mg/dL)	167 (136–186)	177 (144–187)	179 (146–186)	0.7
HDL (mg/dL)	47 (43–55)	44 (40–47)	45 (42–51)	0.2
LDL (mg/dL)	96 (77–114)	108 (79–121)	108 (82–115)	0.6

^a^ Values are presented as median and interquartile range (Q1, Q3).

^b^ Kruskal–Wallis rank sum test.

Regarding metabolic parameters, fasting glucose levels did not show significant differences between groups (p = 0.10); however, serum insulin levels were markedly higher in the CO group (mean = 39 μIU/mL, range = 26–48 μIU/mL) compared to the C group (mean = 8 μIU/mL, range = 7–14 μIU/mL) and the PO group (median = 9 μIU/mL, range = 7–12 μIU/mL) (p < 0.001), confirming compensatory hyperinsulinemia in this group.

No significant differences were observed in total cholesterol (p = 0.7), HDL (p = 0.2), or LDL (p = 0.6), indicating that dyslipidemia was not a predominant factor in the metabolic alterations observed in subjects with clinical obesity and insulin resistance.

**FIGURE 1:**
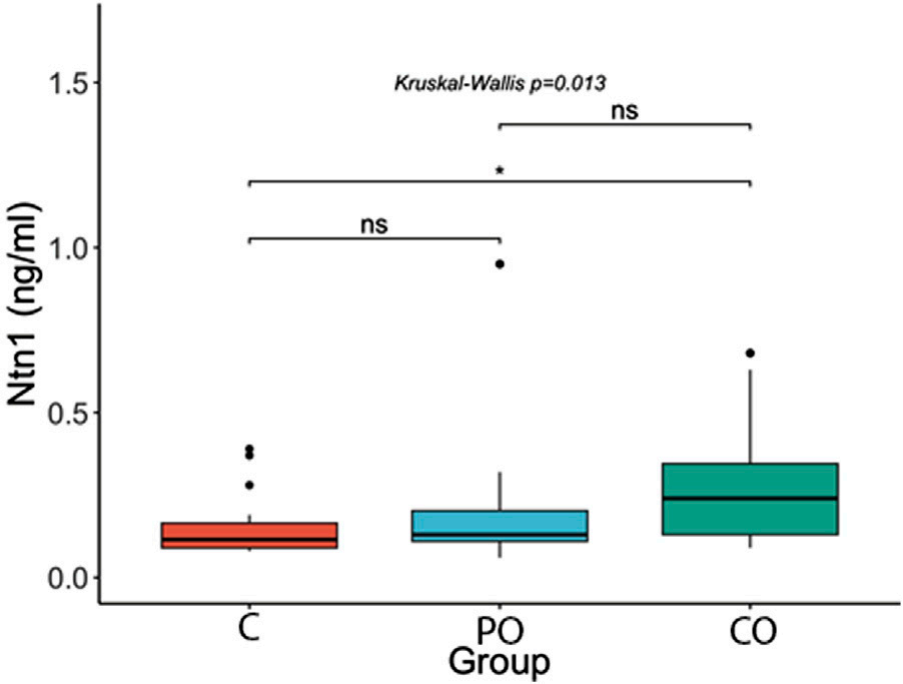
Comparison of Netrin-1 (Ntn1) serum levels. ****: p-value ≤0.0001; ***: p-value ≤0.001; **: p-value ≤0.01; *: p-value ≤0.05; ns = not significant.

**FIGURE 2:**
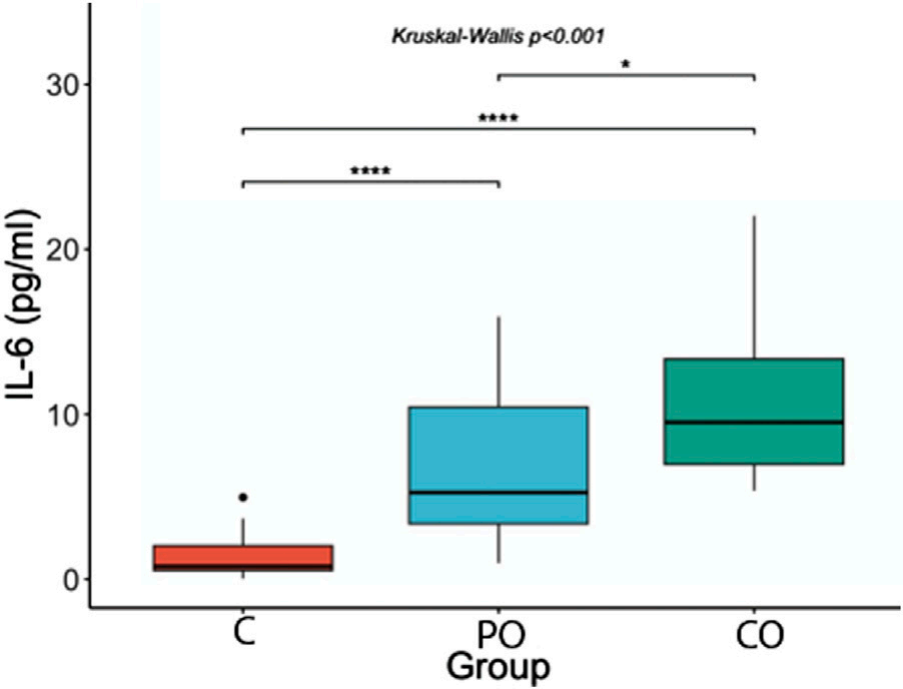
Comparison of IL-6 serum levels. ****: p-value ≤0.0001; ***: p-value ≤0.001; **: p-value ≤0.01; *: p-value ≤0.05; ns = not significant.

**FIGURE 3:**
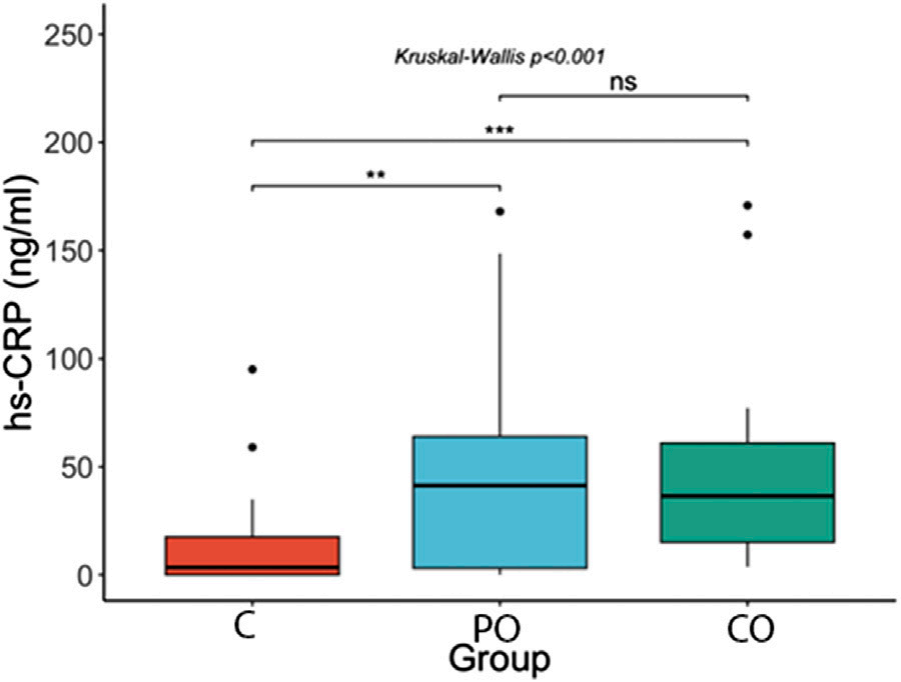
Comparison of hs-CRP serum levels ****: p-value ≤0.0001; ***: p-value ≤0.001; **: p-value ≤0.01; *: p-value ≤0.05; ns = not significant.

Figures 1–3 show the comparison of Netrin-1, IL6 and hs-CRP serum levels between the groups. Netrin-1 showed a statistically significant difference between the CO and the C groups (p ≤ 0.05), while no differences were observed between the PO and C groups, nor between the PO and CO groups. These findings suggest that the increase in Netrin-1 concentration may be more closely associated with the presence of insulin resistance rather than obesity itself.

With regard to IL-6 serum levels, in the *post hoc* analysis we observed that IL-6 concentrations were higher in the CO group (mean = 9.5 pg/mL, range = 6.9–13.9 pg/mL) compared to the PO group (mean = 5.3 pg/mL, range = 3.3–10.5 pg/mL, p-value ≤0.05) and compared with the control group (mean = 0.8 pg/mL, range = 0.5–2.0 pg/mL, p-value <0.0001). Furthermore, the PO group had higher IL-6 serum levels compared with the control group (p-value <0.0001).

As with the results found in the Netrin-1 serum levels, we found no difference in the hs-CRP levels between the CO group (mean = 36 ng/mL, range = 15–62 ng/mL) compared to the PO group (mean = 41 ng/mL, range = 3–69 ng/mL), but we did find it with the control group (median = 3 ng/mL; range = 0–18 ng/mL, p-value <0.001), as well as a difference between the PO and the control group (p-value <0.01).

**FIGURE 4:**
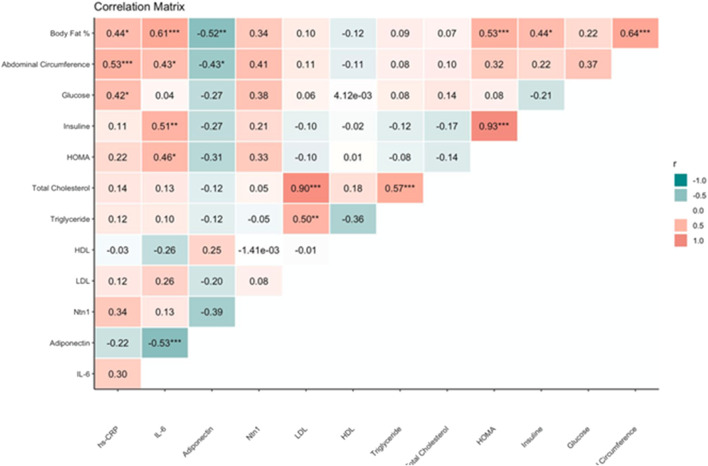
Correlation matrix between anthropometric, metabolic, and inflammatory variables in the study subjects.

Figure 4 shows the correlation matrix between anthropometric, metabolic, and inflammatory variables evaluated in the study.

Existing correlations between adiponectin and IL-6, as well as insulin and IL-6, being negative and positive, were demonstrated with rho = −0.53, p value <0.001 and rho = 0.51, p value <0.01, respectively. Besides, we found a positive correlation between HOMA-IR and body fat % (rho = 0.53, p-value <0.001). No significant correlations between Netrin-1 and the other variables were found.

## Discussion

This study demonstrates that Netrin-1 and adiponectin exhibit opposing serum profiles across a metabolic spectrum—from non-obese individuals to those with preclinical obesity and clinical obesity + insulin resistance. Our findings support the hypothesis that Netrin-1 functions as a pro-inflammatory molecule involved in the retention of macrophages within adipose tissue, contributing to the pathophysiology of obesity-related metabolic dysfunction and insulin resistance. Netrin-1 thus serves not only as a marker of chronic inflammation but potentially as a mechanistic driver, whereas adiponectin acts as a counter-regulatory anti-inflammatory and insulin-sensitizing adipokine (Ramkhelawon et al., 2014; Sharma et al., 2019).

The observed increase in circulating Netrin-1 among participants with obesity and insulin resistance aligns with recent literature showing that Netrin-1 expression in adipose tissue macrophages inhibits their emigration, thereby sustaining local inflammation and exacerbating insulin resistance (Ramkhelawon et al., 2014). For example, Sharma et al. demonstrated that myeloid-specific deletion of Netrin-1 in mice reduced adipocyte hypertrophy, decreased macrophage accumulation, and improved lipid metabolism (Sharma et al., 2019), Similarly, Ramkhelawon et al. showed that Netrin-1 expression promotes macrophage retention in obese adipose tissue, aggravating local inflammation and insulin resistance. Together, these studies suggest a dual role for Netrin-1 as both an initiator and amplifier of chronic adipose inflammation. Our data further support this model, particularly given the significant positive correlation found between netrin-1 and hs-CRP, a well-established marker of systemic inflammation.

Conversely, adiponectin levels declined progressively across groups and were significantly lower in individuals with obesity and insulin resistance. This pattern reinforces adiponectin’s protective role, extensively documented in prior studies. Adiponectin was inversely associated with hs-CRP, body fat percentage, and waist circumference, consistent with literature indicating its anti-inflammatory and metabolic regulatory functions (Geiger et al., 2023; Ziegon and Schlegel, 2022). Ouchi et al. reported that lower adiponectin concentrations are associated with higher CRP levels in both plasma and adipose tissue, mirroring the relationships observed in our study (Ouchi et al., 2011).

The pronounced elevation of Netrin-1 in our insulin-resistant group further supports its potential as an early biomarker of metabolic deterioration. Clinical studies have reported similar associations: Mentxaka et al. demonstrated that netrin-1 and its receptor neogenin-1 are overexpressed in the visceral adipose tissue of obese individuals, promoting a local proinflammatory environment (Mentxaka et al., 2022). Conversely, plasma studies have reported either decreased (Nedeva et al., 2020; Liu et al., 2016) or increased (Yim et al., 2018; Garci et al., 2022) netrin-1 levels in individuals with metabolic dysfunction. These discrepancies likely reflect differences in disease stage, tissue-specific expression, receptor modulation, and systemic inflammatory status, identifying a positive relationship between serum Netrin-1 and inflammatory markers in visceral adipose tissue (Xia et al., 2022), while Shalaby and Nedeva et al. observed increased Netrin-1 levels in individuals with prediabetes and newly diagnosed diabetes (Nedeva et al., 2020; Shalaby et al., 2021).

Nonetheless, conflicting results exist; some studies report reduced Netrin-1 concentrations in patients with impaired fasting glucose or diabetes (Ramkhelawon et al., 2014). These discrepancies may reflect differences in study design, population characteristics, disease staging, or assay sensitivity, as well as the distinction between local tissue expression and systemic circulating levels.

Despite the inverse behavior of these biomarkers, we identified a moderate negative correlation between Netrin-1 and adiponectin (rho ≈ −0.39). While causality cannot be inferred from our cross-sectional data, it is plausible that inflammation-driven upregulation of Netrin-1 suppresses adiponectin synthesis, or conversely, that declining adiponectin levels facilitate Netrin-1 expression through modulation of PPARγ activity and NF-κB signaling (Mentxaka et al., 2022). Specifically, adiponectin enhances PPARγ expression and suppresses NF-κB activation, which may inhibit Netrin-1 expression, whereas reduced adiponectin could relieve this inhibition, promoting netrin-1 upregulation. Alternatively, Netrin-1 and adiponectin may reflect two intersecting but distinct pathways—one promoting macrophage-mediated inflammation, the other exerting insulin-sensitizing and anti-inflammatory effects (Ouchi et al., 2011). This functional interplay warrants further mechanistic exploration.

Further research is needed to elucidate the biological relevance of the Netrin-1– Unc5b signaling pathway and to determine whether resistance mechanisms attenuate the expected effects of elevated circulating Netrin-1. Divergent findings across studies may reflect differences in local versus systemic expression, as circulating levels may not fully capture tissue-specific activity. Investigating concordance between serum Netrin-1 and its expression in adipose tissue could provide critical insights into its role in obesity-associated inflammation (Ramkhelawon et al., 2014; Sharma et al., 2019).

Moreover, the observed inverse correlations between adiponectin and inflammatory markers such as hs-CRP (r = −0.22) and IL-6 (r = −0.53, p < 0.001) reaffirm adiponectin’s anti-inflammatory properties. These findings align with previous studies describing adiponectin’s insulin-sensitizing and anti-inflammatory effects via AMPK activation and suppression of NF-κB signaling pathways. Given the contrasting biological roles of Netrin-1 and adiponectin, this axis represents a promising therapeutic target. Preclinical models demonstrate that neutralization of Netrin-1 or blockade of its receptor Unc5b improves insulin sensitivity and reduces adipose tissue inflammation (Ramkhelawon et al., 2014; Sharma et al., 2019). Simultaneously, interventions known to increase adiponectin levels—such as physical activity, caloric restriction, or thiazolidinedione therapy—could synergistically enhance metabolic outcomes.

In contrast, the anti-inflammatory and insulin-sensitizing effects of adiponectin have been well characterized, including its ability to downregulate pro-inflammatory cytokines, as described by Lawler et al. (2016) and others (Lawler et al., 2016). Reduced adiponectin levels, as observed in our clinical obesity group, likely contribute to the persistence of inflammatory signaling cascades such as NF-κB activation, thereby amplifying metabolic disruption. The inverse trends between adiponectin and both hs-CRP and netrin-1 observed in our study further reinforce this immunometabolic dichotomy.

The cross-sectional nature of our study limits the ability to infer temporal or causal relationships. Longitudinal studies are necessary to clarify the directionality and biological significance of these associations. Additionally, although our sample size was adequate to detect moderate effects, it may not capture more nuanced interactions between biomarkers or subgroup differences.

This study has several limitations. First, the relatively small sample size and single-center recruitment from Guadalajara, Mexico—a predominantly Mexican mestizo population—may limit the generalizability of our findings to other ethnic groups, given known ethnic-specific variations in metabolic biomarkers. Second, the absence of detailed data on dietary intake, physical activity, and other lifestyle factors represents potential unmeasured confounders influencing inflammatory status and adipokine levels. Third, biomarker measurements were performed at a single time point, which may not fully reflect the dynamic nature of metabolic and inflammatory responses. Future multicenter studies involving larger, ethnically diverse populations with longitudinal sampling and comprehensive lifestyle assessments will be essential to validate and extend our results.

Despite these limitations, our findings contribute valuable insights into the molecular mechanisms underlying obesity-associated insulin resistance. We propose that the reciprocal relationship between Netrin-1 and adiponectin merits further investigation, particularly regarding macrophage polarization and chronic low-grade inflammation. Future research should explore the functional consequences of modulating this axis and assess the clinical relevance of Netrin-1 as a biomarker or therapeutic target in metabolic disease. Additionally, combining Netrin-1 and adiponectin measurements may improve diagnostic accuracy and therapeutic monitoring beyond single biomarker approaches.

## Conclusion

Our findings indicate that Netrin-1 and adiponectin exhibit opposing patterns in the context of metabolic inflammation and insulin resistance. Specifically, Netrin-1 levels increase alongside markers of inflammation, whereas adiponectin levels decrease, suggesting a disrupted immunometabolism balance. This contrasting behavior may reflect a mechanistic axis contributing to the progression from metabolically healthy obesity to insulin resistance. However, due to the cross-sectional nature of this study, causal relationships cannot be established. Therefore, longitudinal and interventional studies are warranted to elucidate the temporal dynamics and therapeutic potential of targeting this axis in metabolic disorders.

## Data Availability

The original contributions presented in the study are included in the article/supplementary material, further inquiries can be directed to the corresponding author.
